# Rapid situation & response assessment of diarrhoea outbreak in a coastal district following tropical cyclone AILA in India

**Published:** 2011-04

**Authors:** Samiran Panda, Kamala Kanta Pati, Mihir Kumar Bhattacharya, Hemanta Koley, Sobha Pahari, G. Balakrish Nair

**Affiliations:** *National Institute of Cholera & Enteric Diseases (ICMR), Kolkata, India*; * *Office of the Chief Medical Officer of Health, Purbo Medinipur, India*; ** *Society for Positive Atmosphere & Related Support to HIV/AIDS (SPARSHA), Kolkata, India*

**Keywords:** Antibiotic use, diarrhoea, India, tropical cyclone AILA

## Abstract

**Background & objectives:**

Cyclone AILA hit Indian States on eastern coast on May 25, 2009. An investigation was conducted to examine if AILA was responsible for increased reporting of diarrhoea cases from the district of East-Medinipur in West Bengal. Identifying causative organisms for diarrhoea and assessing their antibiotic susceptibility profile were other objectives.

**Methods:**

Rapid situation and response assessment technique was employed to triangulate primary and secondary data collected through field visits. Prescription audit was also conducted.

**Results:**

Significantly increased occurrence of diarrhoea was observed in June 2009 in two subdivisions namely Haldia and Egra (OR 1.6 and 1.3 respectively; 95% CI 1.52-1.65 and 1.21-1.32 *P*<0.001) considering 2007 as baseline. *Vibrio cholerae* grew from 54 per cent of the stool samples (21/39; 17 *V. cholerae* O1-Ogawa and 4 non-O1-non-O139), confirming a community outbreak of cholera. *Shigella flexneri* 3a was isolated from 5 per cent stool specimens. Increased rate of admission in treatment centres due to diarrhoea in the whole district coincided with the formation of cyclone and showed over two-fold rise compared to the admission recorded 6 days ago. Haldia subdivision had the highest attack rate of 9 per 1000 in the month of June, 2009 whereas for the whole district it was 5 per 1000 in the same month. All the isolates of *V. cholerae* were resistant to ampicillin and furazolidone and sensitive to norfloxacin and azithromycin.

**Interpretation & conclusions:**

Pre-AILA changes in the environment, AILA and seasonality of diarrhoea in the study district interplayed towards increased occurrence of diarrhoea. Continuous tracking of ‘seasonality of diarrhoea in the community with vulnerability assessment of potential hosts’, ‘antibiotic sensitivity profile of the causative microorganisms’, and ‘prescription practice of physicians’ would help appropriate disaster management.

A severe cyclonic storm, AILA crossed West Bengal^1^ coast near Sagar Island at Bay of Bengal between 1330 and 1430 h Indian Standard Time (IST) on May 25, 2009. It continued to move northward. The areas and districts hit hard were either directly on its way or proximally located to the path of the cyclone such as South 24 Parganas, East-Medinipur, Kolkata, Howrah, Hooghly, Burdwan, Birbhum, Bankura and Malda^2^. Deaths and devastations were of varying degree in these districts.

Though India is a disaster-prone country in Asia-Pacific region with an average of 8 major natural calamities a year, the nature of disasters differs in different geographical regions of the country. The eastern coastal areas (Andhra Pradesh, West Bengal, Orissa) are prone to severe floods and cyclones^3^. In 2000-2001, nine districts, 1412 villages and 218180 individuals were impacted by heavy rains and floods in West Bengal and 1320 human lives were lost^4^. Data on impact of such disasters on human health in totality have been sparse^5^. Also developing countries are disproportionately affected by such disasters^6^.

At the behest of the Government of India and State of West Bengal, a post-AILA investigation was conducted in East-Medinipur, one of the coastal districts of the country in June 2009. East-Medinipur (formed following partition of erstwhile Medinipur into two new districts - namely East and West-Medinipur) has 4 sub-divisions; Tamluk, Contai, Egra and Haldia. Sporadic cases of diarrhoea in different parts of the district were being reported since the first week of May, 2009. Following AILA, in the last week of May, 2009 increasing number of diarrhoea cases were reported in parts of East-Medinipur. An expert team from National Institute of Cholera and Enteric Diseases (NICED) was requested to be sent to provide investigation based guidance for prevention and control measures. We report here the rapid situation and response assessment technique employed for investigation in East Medinapur and the findings resulting thereof.

## Material & Methods

The study methodology comprised generating primary data and triangulating these against secondary information collected through field visits. The first field visit was carried out during June 1-3, 2009. Stakeholders comprising district and village administrators and health officials were interviewed. The purpose of this visit was to identify plausible reasons for reported increase in the occurrence of diarrhoea in the district and to find out causative organisms for the same. NICED had the requisite institutional ethics committee clearance to conduct outbreak investigation.

*Study area and population:* During the first field visit one rural hospital (RH, Basulia), one sub-divisional hospital (SDH, Haldia) and one Block Primary Health Centre (BPHC, Nandigram) were covered. Under informed consent, in-patients suffering from diarrhoea or their attendant relatives were administered a brief questionnaire. Rectal swabs from these patients were also collected. The decision about visiting these centres was guided by information on largest number of case-attendance. This information was provided by the district health administration. All cases with diarrhoea admitted within the last 24 h of the investigation day were intended to be covered during investigation; the actual coverage for in-patients achieved was later estimated to be 93 per cent (27/29). During the present cross-sectional study, secondary data were collected for the last three years, and in trend analysis of this data, diarrhoea patients reported from community served as cases and unaffected population as controls.

*Collection, transportation and processing of stool samples:* Rectal swabs were collected from patients during field visit and preserved in Cary Blair’s transport medium. Direct plating of thiosulphate-citrate-bile salts-sucrose (TCBS) agar was carried out as well, whenever possible, with liquid stool specimen collected from the in-patient diarrhoea cases and incubated in a portable system. Presumptive *Vibrio cholerae* isolates formed sucrose fermenting yellow pigmented colonies, which were later found to be oxidase positive and produced an acid slant and acid butt (A/A) without gas formation in Triple sugar Iron medium.

Rectal swabs transported in Cary Blair’s medium to the laboratory, NICED, Kolkata, were plated on to MacConkey agar (Difco, USA) for *Escherichia coli*, Hektoen enteric agar (HEA, Difco, USA) for Shigellae and TCBS agar (Difco, USA) for *V. cholerae*. Physiological and biochemical characterizations of the *V. cholerae* 01 Ogawa strains were performed as described by Sakazaki and Shimada^7^. Polyvalent 01 antiserum and commercially available monoclonal antibodies against each of the antigenic factors A, B, and C of the *V. cholerae* 01 serogroup (Denka Seiken, Tokyo, Japan) were used. *E. coli* was identified using standard morphological and biochemical tests. Confirmed isolates of *E. coli* were examined for presence of heat labile toxin (lt) and heat stable toxin (st) virulence genes by multiplex PCR method^8^. As no bedside microscopy was possible in the reported outbreak situation, identification of any parasite in the stool was not done.

As some of the diarrhoea stool samples sent from the study district in the early weeks of May, 2009 tested positive for *V. cholerae*, at the Bacteriology laboratory of NICED and information was available on antibiotic sensitivity pattern of *V. cholera* isolated from cases admitted to the Infectious Disease (ID) Hospital, Beliaghata in Kolkata during the same month, this information was shared with the health-officials of the district.

*Data collection:* Information on socio-demographic profile of patients, illness history, drinking water source, use of ‘oral rehydration solution’ (ORS) following diarrhoeal attack and treatment provided at the centres was collected. These data and particularly the ones on treatment of diarrhoea cases revealed that prescription audit for cases treated before and after the field visit would generate insight about any change that might be resulting from the present intervention. Prescription audit was undertaken to compare antibiotic use for treating diarrhoea 6 days prior to the first field visit and 6 days after.

*Antibiotic sensitivity testing:* Antibiotic susceptibility testing was performed according to the method of Bauer *et al*^9^ on Mueller-Hinton agar (Difco, USA) by using commercial antibiotic disks (BD BBL ^TM^ Secsi-Disc^TM^, USA). The following disks were used (concentration in μg except where indicated), furazolidone (FX, 100), ampicillin (AM, 10), azithromycin (AZM, 15), amikacin (AN, 30), ciprofloxacin (CIP, 5), co-trimoxazole (Co, 25), chloramphenicol (C, 30), doxycyclin (D, 30), erythromycin (E, 15), gentamicin (GM, 10), kanamycin (K, 30), neomycin (N, 30), nalidixic acid (NA, 30), norfloxacin (NOR, 10), ofloxacin (OFX, 5), streptomycin (S, 10), tetracycline (TE, 30), cefotaxime (CTX, 30) and ceftriaxone (CRO, 30). Bacterial strain, *E. coli* ATCC 25922 was used for quality control limits. The results were interpreted as per the guidelines of the Clinical and Laboratory Standards Institute (CLSI)^10^.

*Statistical methods:* In order to know if AILA had any temporal link with the reported increase of diarrhoea cases from parts of East-Medinipur, chi-square test was done for trend separately for each of the four sub-divisions considering 2007 as baseline. Difference in use of norfloxacin in treating diarrhoea between pre- and post-AILA time reflected through prescription audit and difference in proportion of clinical symptoms suffered by patients in community as opposed to those recruited from treatment centres was examined by chi-square test for proportion. Age of patients recruited from community and from treatment centres was compared by Mann-Whitney U test.

## Results

*Increasing occurrence of diarrhoea after cyclone:* Post-AILA risk of diarrhoea in June, 2009 compared to June 2007 (baseline) was estimated to be 1.6 (95% CI 1.52 to 1.65) and 1.3 (95% CI 1.21 to 1.32) times higher for the sub-divisions of Haldia and Egra respectively (Table). The other two subdivisions namely Contai and Tamluk did not show such heightened risk.

**Table:** Sub-division-wise trend of occurrence of diarrhoea in June during 2007-2009

Sub-divisions in East- Medinipur	Diarrhoea cases No. (attack rate per 1000)	Population not affected	Odds Ratio (OR) (95% CI)
Tamluk 2007	12492 (7.8)	1585008	Reference
2008	3920 (2.4)	1614382	0.31 (0.3-0.32)
2009	5358 (3.2)	1634008	0.42 (0.4-0.43)
Haldia 2007	3773 (5.6)	664666	Reference
2008	2346 (3.5)	66765	0.62 (0.59-0.65)
2009	5988 (8.9)	664901	1.59* (1.52-1.65)
Contai 2007	8735 (7.2)	1208984	Reference
2008	6894 (5.6)	1226034	0.78 (0.76-0.8)
2009	5192 (4.1)	1243084	0.58 (0.56-0.6)
Egra 2007	3631 (4.2)	865702	Reference
2008	2233 (2.3)	950143	0.56 (0.53- 0.59)
2009	4624 (5.3)	868709	1.27* (1.21-1.32)

* *P*<0.001; CI, confidence interval

Day-wise report of 24 h admission of diarrhoea case load at 12 noon at the indoor of health centres and hospitals revealed that the total admission on the day of formation of Cyclone (May 24, 2009) and the next day when it hit the coast increased by more than two-fold compared to the admission scenario 6 days ago for the whole district of East-Medinipur. While the number of total admission due to diarrhoea in the district was 106 on May 18, it was 254 on May 24 and 222 on May 25 following which it started declining.

**Fig. 1:**
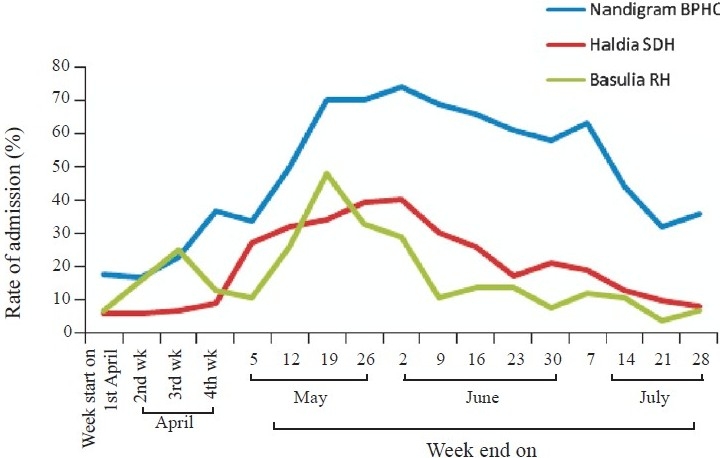
Rate (%) of admission due to diarrhoea in treatment centres. RH, rural hospital; SDH, sub-divisional hospital; BPHC, Block primary health centre.

*Indoor admission and patient characteristics:* Indoor admission rate due to diarrhoea in the three treatment centres visited for investigation under Haldia sub-division revealed an upward trend starting in the second week of May (Fig. 1). While admission due to diarrhoea in the 1^st^ wk of April in Basulia Rural Hospital was 7 per cent (4/61) it reached a noticeable height of 48 per cent (53/110) and 33 per cent (44/134) during the 3^rd^ (13^th^ to 19^th^ May) and 4^th^ wk of May (20^th^ to 26^th^ May) respectively. Subsequently, the rate came down to 29 per cent (48/164) after AILA (week between 27^th^ May and 2^nd^ June), and further down to 11 per cent (15/136) in the next week. Diarrhoea admission rate showed similar trends in Nandigram Block Primary health centre (18%; 11/60 in the 1^st^ wk of April, and 70%; 94/135 during the 4^th^ wk of May) and Haldia sub-divisional hospital (6%; 12/196 and 39%; 105/271 respectively). Attack rate of diarrhoea in the month of May (20662/4429864) and June (21162/4429864) in the district of East-Medinipur reached an all time high of 5 per 1000 compared to that in other months of 2009. Haldia, the port subdivision on the coast, had the highest diarrhoea attack rate of 9 per 1000 in June 2009 (Table).

Sixty four per cent of the respondents contacted at treatment centres as well as in the community (25/39) had acute attacks of watery diarrhoea on the day of interview. Information collected from 39 patients during the first field visit indicated striking similarity in the illness experienced across gender and age; 18 were males (46%) and 21 (54%) females. Twenty seven of 39 patients (69%) were from treatment centres while the rest were at their residence. While three patients were below one year of age, 6 belonged to the age group 1 to 5 yr (mean 2 ± 1 yr; median 2 yr; range 1.5-3.5 yr) and 17 to the age group >5 to <18 yr (mean 9 ± 3 yr; median 9 yr; range 6-14 yr). Mean age of 13 patients who were adults (e18 yr) was 33 ± 1 yr (median 30; range 18-55 yr).

Mann-Whitney test revealed that the distribution of age between patients recruited from community (n=12) and those from treatment centres (n=27) was not significantly different. The groups were also similar in terms of clinical features; watery diarrhoea being present in two third of them (8/12; 67% and 17/27; 63%) and cough being present in one third (4/12; 33% and 8/27; 30%). Vomiting (6/12; 50% and 7/27; 26% p=0.1) and fever (2/12; 17% and 3/27; 11%) as one of the current symptoms also did not differ significantly between these two groups.

*Illness profile of in-patients:* All 27 diarrhoea patients except one at treatment centres, had passed stool three times or more in a day while getting admitted. Only one of the 27 patients reported passing blood with stool and the rest had acute watery diarrhoea. The mean frequency of passage of stool per day on the day of interview was 6 times (median 6; minimum 1 and maximum 15) and a little more than one fourth of the admitted cases (7/27) reported vomiting as one of the current symptoms – the frequency ranging from 1 to 3 times a day. About 80 per cent (21/27) of the patients during interview had some dehydration and 7 per cent had severe dehydration on examination while the rest had no dehydration.

*Illness in the family, drinking water practice and use of ORS:* One third (12/39; 32%) of the diarrhoea cases reported that at least another family member and 66 per cent (25/39) said that others in the community had been suffering from similar illness. Majority of the interviewees (37/39; 95%) used tube well as source of drinking water and only one tenth either regularly used chlorination, filtering or boiling as water purification method. Earthenware pot (16/39; 41%), pitcher made of aluminium (9/39; 23%) or brass (6/19; 15%) were the vessels where most of the respondents regularly preserved drinking water and all without exception used a lid to cover the mouths of these vessels. Only 26 per cent (10/39) of the patients suffering from diarrhoea reportedly used ORS following attacks of diarrhoea.

*Antibiotic use pattern revealed through prescription audit:* Use of multiple antibiotics to treat cases of watery diarrhoea was noticed from in-patient records. Prescriptions of patients admitted within the past 24 h period at two different time points (6 days prior and 6 days after field visit) were audited. Eighty pre-field-visit and 47 post-field-visit prescriptions were examined, revealing that all in-patients received antibiotic and more than 40 per cent of them actually received 2 and 10 per cent had received 3 or more antibiotics. Injection antibiotic use was recorded in 93 per cent (74/80) of pre-field-visit and 79 per cent (37/47) of post-field visit prescriptions. Ciprofloxacin, gentamicin, cefotaxime, ceftraixone and amikacin were among the injection antibiotics used.

During the first field visit, 21 of 27 (78%) in-patient diarrhoea cases were on antibiotics, of whom three were prescribed a combination of 3 antibiotics (ciprofloxacin, metronidazole and tetracycline). Six in-patients (29%) were receiving a combination of two antibiotics (mostly ciprofloxacin with metronidazole; or combination of ciprofloxacin with co-trimoxazole or tetracycline) and the remaining were given one antibiotic. Norfloxacin use increased significantly after we disseminated laboratory findings to health-administrators (2/80; 3% to 26/47; 55% χ^2^ 48; *P*<0.001).

**Fig. 2:**
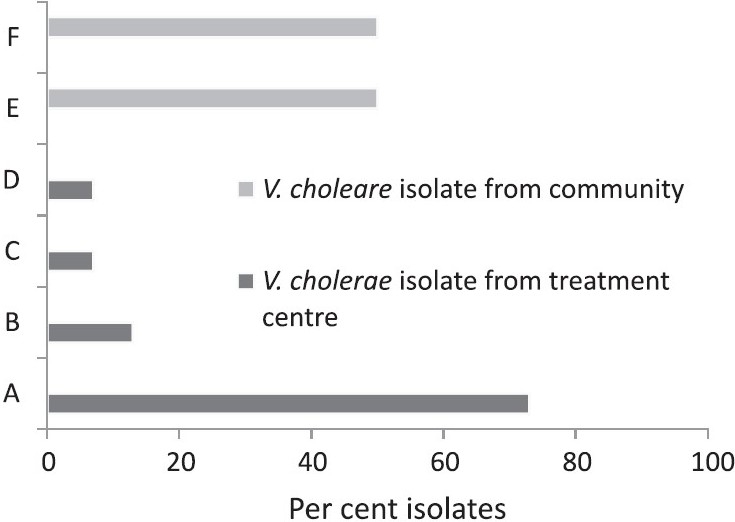
Antibiotic resistance profile of *V. cholerae* isolates of east-Medinipur. A, resistance to AM, Co, FX& NA (11/15); B, resistance to AM, Co, FX(2/15); C, resistance to AM, FX, NA (1/15); D, resistance to AM, Co, FX, NA, C, E, CIP, OFX, GM, K & TE (1/15); E, resistance to AM, Co, NA (3/6); and F, resistance to AM, Co, NA & TE (3/6).

*Stool culture and antibiotic sensitivity results:* *V. cholerae* was recovered from 54 per cent of the stool samples (21/39; 17 *V. cholerae* serogroup O1 Ogawa and 4 non-O1-non-O139), and *Shigella flexneri* 3a from 2 (5%). Seven isolates of *E. coli* (6 from treatment centres and 1 from community) were identified (18%) of which none was found to be toxigenic. All the isolates of *V. cholerae* were sensitive to norfloxacin and azithromycin. Resistance to multiple antibiotics was noted in the isolates of *V. cholerae* cultured from stool samples collected from treatment centres as well as from community (Fig. 2). Both the isolates of *Shigella flexneri* were resistant to furazolidone, chloramphenicol, co-trimoxazole, ciprofloxacin, norfloxacin, ofloxacin, nalidixic acid, streptomycin and tetracycline.

## Discussion

Increase in diarrhoeal diseases following natural disasters of various kinds has been recognised in both developed and developing country settings^6^,^11^. Contamination of the source of drinking water used by the population due to disaster has been responsible for some of these incidents as witnessed in post-tsunami Aceh Province of Indonesia in December 2004^12^ and Car Nicobar Island of India in 2007^13^. Post-disaster diarrhoea has also been recorded in evacuees-camp where the challenge is to properly equip such camps providing shelter to the people struck by disasters. Outbreak of watery diarrhoea in Muzaffarbad, Pakistan following the 2005 earthquake^14^ exemplifies such challenges. Once a diarrhoeal outbreak takes place in a crowded setting, the challenge is to manage and contain the further spread of it as was recognised after Hurricane Katrina in 2005^15^ and Rita in the United States^16^.

In the present outbreak the factors- seasonality and natural calamity interplayed. AILA being associated with a short period of heavy rainfall amplified the scope of spread of diarrhoeal diseases. It is worth noting that although *V. cholerae* is a water-borne pathogen, it is able to spread by direct faecal-oral transmission through improper food handling and the organism acquires a transient hyper-infectious phenotype after passage through the human intestine^17^,^18^, which could contribute to its epidemic spread^19^. It has also been indicated that for every overt case of cholera in the community asymptomatic and mild diarrhoea causing infections by *V. cholerae* could range from 3 to 100^20^. During the present study, one third of the diarrhoea cases, majority of which were cholera, reported at least another family member and 66 per cent said that other community-members had been suffering from similar illness. This affirmed on-going effective transmission of cholera during post-AILA in community-setting.

Two studies – one conducted in children^21^ and the other in adults^22^ in the neighbouring country of Bangladesh during 2000-2004 clearly demonstrated advantage of treating cholera with single dose of azithromycin (1 g dose - two 500-mg tablets for adults and 20 mg/kg body weight of children with upper ceiling of 1 g) compared to the multiple dose treatment with erythromycin or single dose ciprofloxacin respectively. In the present study also all *V. cholerae* isolates were found to be sensitive to azithromycin and norfloxacin and we developed and disseminated a treatment guideline accordingly.

In conclusion, the rapid situation and response assessment technique proved to be the right public health approach in investigating post-disaster diarrhoea outbreak situation. Engagement of the local stakeholders including district administration and key public health officials at every stage of investigation starting from planning through execution may play a pivotal role.
